# Characterization of the physicochemical composition of anaerobically digested (digestate) high throughput red meat abattoir waste in South Africa and the determination of its quality as a potential biofertilizer

**DOI:** 10.1016/j.heliyon.2023.e21647

**Published:** 2023-11-03

**Authors:** Dikonketso Shirleymay Matjuda, Memory Tekere, Mary-Jane Thaela-Chimuka

**Affiliations:** a Agricultural Research Council-Animal Production (ARC-AP), Department of Microbiology and Biotechnology, Old Olifantsfontein Road, Private Bag X2, Irene, 0062, South Africa; b Department of Environmental Science, College of Agriculture and Environmental Sciences (CAES), University of South Africa (UNISA), P.O. Box 392, Florida, 1710, South Africa; c Agricultural Research Council-Animal Production (ARC-AP), Animal Nutrition and Aquaculture, Old Olifantsfontein Road, Private Bag X2, Irene, 0062, South Africa; d School of Animal, Plant and Environmental Sciences, University of the Witwatersrand, Private bag 3, Wits, Braamfontein, 2050, Johannesburg, South Africa

**Keywords:** Digestate, biofertilizer, Red meat, Abattoir waste, Anaerobic digestion, High throughput

## Abstract

Anaerobic digestion as a treatment option for waste produced in high throughput red meat abattoirs in South Africa is now gaining interest in both private and government sectors. The resultant digested slurry (digestate) is currently being regarded as waste despite its nutritional value for soil and plants which can be harnessed if digestate is utilized as biofertilizer to ensure nutrient cycling. The study investigated the physicochemical and microbial characteristics of digestate emanating from anaerobic digestion of red meat abattoir waste in South Africa, as well as evaluating its potential use as biofertilizer. The pH, total solids, volatile solids, chemical oxygen demand, electrical conductivity, total volatile fatty acids and chemical composition were determined using standard methods. Microbial analyses were determined according to the serial dilution method (10^1^- 10^10^). The results were benchmarked with Public Available Specifications (PAS) 110 standards for quality control of digestate intended to be used as biofertilizer for agricultural purposes. Results for pH, total solids, electrical conductivity, chemical oxygen demand, and total volatile fatty acids fell within the required PAS110 standard which requires standard limits of 6.5–9, 30 %–50 %, <1500 mg/L, <3000 μS/cm, and 0.43 COD/g VS respectively. Moisture content in all red meat abattoir digestate ranged from 92.05 ± 0.5 % to 95.49 ± 0.38 % and did not meet the required limit of <35 %. *E. coli* in untreated cattle and pig abattoir digestate were 1023 ± 35 cfu/mL and 1068 ± 51 cfu/mL, respectively, and also did not meet the required standard limit of <1000 cfu/mL. Chemical composition showed that abattoir digestate was abundant in both macronutrients and micronutrients, and heavy metal concentrations in all digestate samples fell within the PAS 110. In conclusion, abattoir digestate was observed to be highly abundant in nutrients essential for soil health and plant growth, and mostly met the required EU PAS110 standard for utilization as biofertilizer in agricultural land.

## 1 Introduction

Waste management concerns in red meat abattoirs have been increasing in the past decades due to the rise in population in both developing and developed countries [1]. The increase in population has resulted in increase in the number of animals being slaughtered in abattoirs in order to meet the growing food demands, thus resulting in large quantities of waste being produced during slaughtering activities [1,2]. South Africa is a major red meat exporter in Africa with red meat high throughput abattoirs slaughtering over millions of cattle, sheep, and pigs per annum [2,3]. As waste generated in abattoirs is considered hazardous to both human and the environment due to heavy metals, pathogens and other toxins, various options in treating this waste has been investigated including anaerobic digestion for biogas production [3].

Anaerobic digestion (AD) technology for biowaste treatment has attracted more interest from governments in developing countries including South Africa in recent years when compared to other treatment methods for organic waste [1]. In addition to biogas, the AD process produces digestate which is a partially decomposed by-product rich in plant nutrients [[1], [2], [3], [4]]. As a potential replacement of synthetic fertilizers in agriculture and farmlands, the digestate has strong biofertilizer and organic amendment characteristics [5]. Because of its unique chemical composition and concentration of essential plant nutrients, particularly soluble potassium and N-reduced forms, digestate has the capacity to act as a soil conditioner enhancing physical properties and release soluble plant nutrients [1,6,7].

These soluble plant nutrients contained in digestate enhances its valorization as biofertilizer or soil amendments to help against the increased demand for synthetic chemical fertilizers and may reduce the financial and environmental expenses of chemical fertilizer synthesis and waste management [6,7]. Even though, digestate has been labelled in literature as a nutrient-rich product, the significant proportion of its known undesirable components, such as heavy metals, phytotoxic substances, pathogenic bacteria, odour-emitting substances, an unstable nutrient content, and an excess of inorganic N forms, can negatively affect the environment [1,8]. The characteristics of digestate dependent on several factors that are interlinked, including feedstock composition, inoculum used, AD operation condition i.e., hydraulic retention time (HRT), organic loading rate (OLR), mixing, temperature, pH, and digestate management techniques and NH_4_^+^-N content [9,10].

Higher NH_4_^+^-N content is of great importance in a fertilizer, as it is immediately available to the plant and digestates have been reported to contain high proportion of plant readily available NH_4_^+^-N in mineral form [2,[10], [11], [12], [13], [14]]. Digestates with C/N ratios of 15–20 range are considered to be safe for application on agricultural land without further treatment [2,5,15]. Digestate's biological stability is a quality that should be taken into account when determining whether or not to use it in agriculture as an organic amendment to balance the humus content of the soil [5,[16], [17], [18]]. Utilizing digestate as biofertilizer versus untreated organic materials ensures recycling of nutrients harvested during AD of organic waste which could have been lost when the waste was disposed using other disposal methods [18]. The degradation efficiency of organic waste during AD is of great importance to ensure optimum nutrient recycling and sanitary levels quality of the digestate as biofertilizer [19].

The digestate quality and its potential agronomic use as biofertilizer depends on the following factors; segregation and loss of nutrients in the feedstock; digestate storage structures; the efficiency of feedstock's pre-treatment systems and/or digestate treatment [19,20]. As abattoir waste is classified under category 3 waste (hazardous waste), the South African government requires a mandatory pre-treatment of abattoir feedstock waste (as a sanitary precaution or requirement) either by sterilization or pasteurization prior to all biological treatments including AD [2,4,10,13,14,21,22]. This is so that on-site treatment, including AD, can be encouraged because the South African government currently forbids the dumping of abattoir slaughter waste in landfills due to the high expense of processing the waste. However, the South African government continues to classify the digestate generated by abattoir biogas facilities as environmentally hazardous. This is because there could be negative consequences on the soil's quality, human and animal health, due to excessive COD levels, the presence of pathogens, the physical blockage of the soil caused by fat deposition, and high content levels of heavy metals [22,23]. This mandatory pre-treatment of red meat abattoir waste in biogas plants and the impacts it will pose not only on biogas production but also on digestate quality need to be investigated in South Africa to add value and to generate new knowledge to the red meat industry as well as for policy making for the local government.

Studies on comprehensive characterisation of the digestate from anaerobic digestion of red meat solid abattoir waste are limited worldwide and are non-existent in Africa [22,23]. Furthermore to our knowledge no studies on characterizing AD red meat abattoir slaughter waste (digestate) and its quality as potential biofertilizer have been done in South Africa. While characteristics and biofertilizer quality of digestate from AD food waste, manure, agricultural was and waste wastewater both from abattoirs and municipal waste has been extensively researched [[23], [24], [25], [26], [27], [28]]. Bearing all this in mind this study's objective was to determine the physicochemical characteristics of red meat abattoir derived digestate and to evaluate its safety quality as potential biofertilizer. The study intends to fill literature gap by adding knowledge on characteristics of digestate from AD red meat abattoir solid waste and its sanitary quality as potential biofertilizer. It is also critical to develop efficient post-treatment and valorization procedures during soil amendment and land application [10,24,25].

## 2 Materials and methods

### 2.1 Sample collection and digestate sample preparation

About 180 abattoir slaughter waste samples were collected from 45 high throughput red meat abattoirs across South Africa. High-throughput abattoirs slaughtering more than 250 animals per week obtained from feedlots and commercial farmers were selected to partake in this study. Abattoirs were also selected according to the type (red meat only: cattle, sheep and pig) of animals they slaughter daily and consent from Abattoir management to partake in the study.

Abattoir slaughter waste such as blood, condemned, and rumen contents were collected for a period of a year from March 2021–February 2022 and the sampling was repeated 4 times during the sampling period at each sampling points. Samples were transported on ice from sampling points to the laboratory and then kept in the freezer at −20 °C till further analysis. The different wastes components were mixed and blended to a size of about 2 mm and pre-treated using pasteurization (70 °C for 1 h) and sterilization (133 °C at 3 bar for 15 min) prior to AD in according to the Regulation (EC) No. 1069/2009. Abattoir waste that was not subjected to thermal pre-treatment is referred to as untreated abattoir samples in this study. All red meat abattoir waste samples (untreated, pasteurized, and sterilized) were anaerobically digested using the Automatic Methane Potential Test System (AMPTS) II in 500 mL digester bottles placed in water bath (with temperature of 37 °C) with a hydraulic retention time of 40 days following manufactures protocol. At the end of digestion, about 200 mL of digestate samples (180) (resultant slurry from digested red meat abattoir) were sent to Agricultural Research Council (ARC) - Institute for Soil Climate and Water laboratory for analysis of micronutrients, macronutrients, and heavy metals composition. The method for drying digestate was adopted from Ref. [29] with amendments. Briefly, digestate samples were dried to reached 90 % solid concentration at 25 °C for seven days in an incubator, then shredded in a blender after then they were milled in a 1 mm mesh and analyzed for their chemical properties. Drying of digestate was also adopted from the recommendation stipulated by Ref. [30] for ensuring a mature and stabilize digestate as well as to meet the requirements for a hygienic and transportable product. The physical and microbial characteristics of digestate were analyzed using fresh digestate samples.

### 2.2 Analysis of red meat abattoir digestate physicochemical characteristics

The following physiochemical properties of digestate were analyzed: pH, total solids (TS), volatile solid (VS) and ash were determined using standard methods [31] and according to Ref. [32]. Chemical oxygen demand (COD), Ammonia-nitrogen (NH_4_^+^-N), Carbon (C) concentrations were determined according to the standard methods for examination of water and wastewater [31]. Nitrogen (N) concentration was determined using the Kjeldahl method according to Standard method 351.2 [31]. All physicochemical characteristics analyses were done in triplicates.

To determine the TS, the weight of the empty crucible was recorded, then, approximately 5g of the sample were filled into the crucible and the weight of the filled crucible was recorded. The samples were placed in the incubator at 105 °C and left to dry in a desiccator overnight. The weight of the crucible with the dried sample was then recorded and the TS was calculated according to equation (1) as adopted from Ref. [33]:

(1) $$TS\;\left(\%\right)=100\times \frac{m3-m1}{m2-m1}$$

Where:

TS = Total Solids (%)

*m*1 = weight of empty crucible (g)

*m*2 = weight of crucible + sample (g)

*m*3 = weight of crucible + sample after drying (g)

To determine the ash contents, dried samples were calcined in the muffle furnace for 3 h at 550 °C. After the calcination, the hot crucibles were cooled in a desiccator and after the cooling they were reweighed, and results were recorded as ash contents. Ash was calculated in accordance to equation (2) as follows:

(2) $$Ash\;\left(\%\right)=100\times \frac{m3-m4}{m2-m1}$$

Ash = Ash contents (%)

*m*1 = weight of empty crucible (g)

*m*2 = weight of crucible + sample (g)

*m*3 = weight of crucible + sample after drying (g)

*m*4 = weight of crucible + sample after calcination (g)

Volatile solids were calculated according to equation (3) adopted from Refs. [33,34]:

(3) $$VS\;\left(\%\right)=100\times \frac{Ash}{TS}$$

Carbon concentration in samples was analyzed spectrophotometrically based on the standard method 10128 [31]. About 10 mL of sample was used in the digestion, placing the sample mixed with the reactants into the DRB 200 reactor for 2 h at 105 °C. After heating, the vials were carefully removed from the reactor and allowed to cool for 1 h in a test tube rack. The absorbance was measured spectrophotometrically at 495 nm using HACH DR 500.

The pH was read using PHC101 probe and the pH meter was calibrated using buffers of pH 4.0, 7.0 and 10.0 (Whitehead Scientific (Pty) Ltd). The pH reading was taken in triplicates using 20 mL of the digestate sample and the PHC101 probe was rinsed with deionized water in between the readings. The N concentration was determined using the Total Kjeldahl Nitrogen method according to Standard method 351.2 [31].

Ammonium-nitrogen was analyzed colometrically using method as described by Ref. [33]. Briefly, the 10 mL samples were centrifuged for 10 min at 10 °C and 10,000×*g* and the supernatant was diluted corresponding to the measuring range of the photometer (1:1000). About 2.5 mL of the dilution were placed in a cuvette and three drops of mineral stabilizer, three drops of polyvinyl alcohol, and 1 mL of Nessler's reagent were added prior to measuring NH_4_^+^-N concentrations. The mixture was mixed by swirling and mixture place on bench for 2 min to complete reaction. NH_4_+-N concentrations were read using spectrophotometer (HACH DR 500) at absorbance of 495 nm and calculated against standard solutions of known concentrations.

Determination of COD was done using standard method 8000 (reaction digestion method) [31]. Results were read using spectrophotometer (HACH DR 500) with the high COD range test program. Analysis for the other chemical parameter such as phosphorus (P), potassium (K), and sulphur (S) was performed according to the standard methods for examination of water and wastewater [31]. Total Organic Carbon (TOC) was analyzed using Standard method 9060A [31]. Concentrations of heavy metals such as Cu, Zn, Mn, Pb, Ni, Cr, Cd, As, Fe, Hg were analyzed spectrophotometrically using standard methods for examination of water and wastewater [31]. Results were measured at 495 nm using spectrophotometer (HACH DR 500) and samples were analyzed in triplicates.

### 2.3 Determination of analyses of VFA in digestate

The method used to analyse VFA was adopted from Ref. [35] as follows: Individual volatile fatty acids (lactic, formic, acetic, propionic, n-butyric, *iso*-butyric, *n*-valeric, *iso*-valeric and caproic) were quantified with a high-performance liquid chromatography (HPLC) using a Finnigan Surveyor chromatograph (Thermo Scientific, San Jose, USA) coupled with an Aminex HPX 87H column (Bio-Rad, Hercules, USA) and a refractive index detector. Separation during the HPLC tests was performed using sulphuric acid (5 mmol/dm^3^) as a mobile phase, which was applied at a flow rate of 0.6 cm^3^/min.

### 2.4 Analysis for the detection of coliforms, Listeria and Salmonella in digestate

The media utilized for the analysis of coliforms and *Salmonella* were, Violet Red Bile Agar (coliforms), McBride *Listeria* Agar (*Listeria* sp.) Brilliant Green Agar (*Salmonella* sp.). Serial dilution whereby one mL of the fresh digestate sample was used in conducting the serial dilution of 10^−1^ up to 10^−8^ on a sterile 0.9 % (w/v) saline solution was done [36,37]. Pour plate method was used whereby 1 mL of each dilution was placed in aliquots of 15 mL of agar and mixed by swirling the petri-dish side by side. Agars were left to solidify in room temperature and incubated at 37 °C for 48 h. Colony counts were performed and results were expressed in colony forming unite per millilitre (cfu/mL).

### 2.5 Statistical analysis

All analysis in this study were performed in triplicates. Statistical significance of the results was confirmed by two-way ANOVA (P < 0.05). The differences of each treatment were studied by *t*-test. For statistical analysis, GENSTAT statistical program was used. Biofertilizer potential of digestate was determined by benchmarking the results obtained in this study with European regulation PAS 110 [38], which stipulates the required physical, chemical and microbial limits for quality control of digestate intended to be used as biofertilizer in agricultural fields to safeguard against environmental contamination, human health and animal health.

## 3 Results and discussion

### 3.1 Results for the physicochemical characteristics analysis of South African red meat abattoir digestate

As required by PAS 110 [38] fertilizer regulations, the characterization of digestate in this study was carried out in compliance with quality requirements for safe and effective usage in agriculture as biofertilizer. Table 1 shows the physicochemical characteristics results of the South African high throughput red meat abattoir digestate from analyzed samples and PAS 110 [38] used as benchmark for quality standard. The table presents the mean and standard deviation of 20 digestates sample for each represented digested abattoir slaughter waste (e.g. 20 samples for untreated cattle waste, 20 samples for pasteurized cattle waste, 20 samples for sterilized cattle waste etc.) The PAS 110 fertilizer regulation aims to provide a standardized regulatory framework in accordance with the circular economy concept, so that the use of digestate as biofertilizer obtained from biogas plants treating various organic waste including abattoir slaughter waste can be encouraged [5,39].

**Table 1:** The physicochemical characteristics of untreated, sterilized, and pasteurized cattle, sheep, and pig anaerobically digested high throughput red meat abattoir waste analyzed at the end of AD process. Results are means ± standard deviation of 20 digested sample per red meat animal type for untreated, sterilized and pasteurized red meat abattoir digestate and results were benchmarked with PAS110 for quality control of digestate.

Parameter	Unit	Results (mean ± SD)									PAS 110
		CSW (untreated)	CSW (sterilized)	CSW (Pasteurized)	SSW (untreated)	SSW (sterilized)	SSW (pasteurized)	PSW (untreated)	PSW (sterilized)	PSW (pasteurized)	Upper Limits
pH		7.86 ± 0.11	8.01 ± 0.03	8.11 ± 0.09	7.91 ± 0.07	8.07 ± 0.05	7.99 ± 0.09	7.83 ± 0.06	7.91 ± 0.10	7.88 ± 0.07	6.5–9
VS	%	4.67 ± 0.58	3.88 ± 0.49	3.12 ± 0.26	4.19 ± 0.38	2.94 ± 0.55	1.69 ± 0.49	4.16 ± 0.35	3.02 ± 0.43	2.33 ± 0.59	–
VS/TS ration		0.56 ± 0.08	0.55 ± 0.01	0.38 ± 0.03	0.61 ± 0.06	0.40.±0.08	0.37 ± 0.07	0.57 ± 0.04	0.44 ± 0.06	0.39 ± 0.05	NA
Moisture	%	91.61 ± 0.56	92.95 ± 0.48	92.05 ± 0.85	93.13 ± 0.66	95.41 ± 0.44	95.49 ± 0.38	92.72 ± 0.51	93.28 ± 0.47	94.16 ± 0.63	<35 %
TS	%	8.37 ± 0.61	7.05 ± 0.48	7.9 ± 0.58	6.87 ± 0.74	4.59 ± 0.81	4.51 ± 0.72	7.28 ± 0.92	6.72 ± 0.63	5.84 ± 0.56	30 %–50 %
COD	mg/L	3724.61 ± 301.75	2048.71 ± 282.65	1441.35 ± 178.08	3873.41 ± 399.17	3128.35 ± 243.11	2657.66 ± 328.15	3140.06 ± 264.72	1813.19 ± 418.31	2061.13 ± 342.09	<1500 mg/L
NH_4_^+^-N	% TKN	51.33 ± 2.01	63.41 ± 3.51	67.09 ± 2.94	54.17 ± 1.84	63.61 ± 2.66	66.37 ± 4.37	50.09 ± 2.19	61.22 ± 2.55	57.59 ± 23.28	–
TKN	g/Kg DM	47.32 ± 2.03	53.26 ± 3.56	57.33 ± 2.36	49.95 ± 1.38	55.61 ± 3.09	57.18 ± 2.67	48.73 ± 2.43	56.47 ± 2.35	55.81 ± 2.86	–
EC	μS/cm	2260 ± 65.91	1977 ± 71.33	1759 ± 83.33	2518 ± 54.12	2366 ± 62.57	2110 ± 50.07	3334 ± 35.33	2519 ± 39.05	1677 ± 25.70	<3000 μS/cm
TVFA	mg/L COD	2872.57 ± 531.89	2359.12 ± 498.15	2102.27 ± 398.21	3230.82 ± 421.89	2588.72 ± 387.55	2308.14 ± 277.99	3100.41 ± 367.41	2440.88 ± 356.48	2149.9 ± 386.67	4.3 g COD/g VS

VS = volatile solids; TS = total solids; COD = chemical oxygen demand; EC = electrical conductivity; TVFA = total volatile fatty acid; TKN = total kjeldahl nitrogen; CSW = cattle slaughter waste; SSW = sheep slaughter waste; PSW = pig slaughter waste; NA = not applicable.

Results for pH values for the high throughput red meat abattoir digestate in this study was observed to be in a range of 7.83 ± 0.06 to 8.11 ± 0.09 across analyzed samples, which fell with the recommended range of 6.5–9 by Ref. [38] (Table 1). The results for pH values in abattoir digestate in this study were not significantly different (p < 0.05), thus indicating that thermal pre-treatment of feedstock has no effect on the final pH of digestate. The pH values in this study had a more alkaline characteristic, which similar trends were also observed in previous research conducted by Ref. [21]. The alkaline characteristic of abattoir digestate observed in this study can be attributed to the degradation of VFAs and ammonia during AD [11,40]. The alkaline nature of digestate favor its use as a biofertilizer as most agricultural crops grow best in slightly alkaline soils [40].

The results for TS, VS, and moisture in this study ranged from 4.51 ± 0.72 to 8.37 ± 0.61, 1.39 ± 0.55 to 4.67 ± 0.58, and 92.05 ± 0.5 to 95.49 ± 0.38 respectively across analyzed digestate samples (Table 1). The TS, VS, and moisture results for cattle and sheep abattoir digested waste differed significantly (p < 0.05) between untreated and pretreated samples and there were no significant difference (p < 0.05) observed amongst sterilized and pasteurized cattle and sheep digestate samples. Results for TS, VS, and moisture in pig abattoir digestate differed significantly between untreated and pasteurized samples (p < 0.05), and the results observed between sterilized and pasteurized pig abattoir digestate samples and between untreated and sterilized samples did not differ significantly (p < 0.05). TS is a very important parameter as it determines the state on potential and practical use of digestate as biofertilizer or for soil amendment. TS values in a range of 3–30 % in digestate has been reported to favor its use as biofertilizer as it could assist in increasing organic matter (OM) in soils low in humus and assist in the stabilization and sequestration of soil organic carbon (SOC) [41,42]. The results obtained in this study were observed to fall within the recommended PAS 110 [38] range.

The VS/TS ratio has been considered as an indicator variable of stability in digestates. Studies by Refs. [43,44] indicated a stability limit range of 0.37–0.77 VS/TS ration in digestates favors its valorization as a biofertilizer than soil amendment. The VS/TS ration values observed in this study ranged from 0.37 ± 0.07–0.61 ± 0.06 across analyzed digestate samples (Table 1). The observed results indicates that digestate in this study followed similar trends as reported by Refs. [43,44] thus proving that AD red meat abattoir waste used as feedstock for biogas production pretreated and untreated has the ability to produce stable digestate thus favoring its use as biofertilizer. The VS/TS ratio of digestate samples in this study was also observed to fall within the recommended ranges described by Ref. [38] of a stable biofertilizer. In accordance to Refs. [43,44] pretreatment has the ability to increase the stability of digestate further by reducing the VS/TS ration and similar trends were observed in this study as values of pasteurized and sterilized samples were lower as compared to those of untreated digestate samples. Pasteurized digestate samples were observed to be more stable as compared to control and sterilized digestate samples in this study which could be attributed to the temperature variation which impacted on solid components of the feedstock prior to AD.

The moisture contents in this study in all digestate samples exceeded the recommended limit of <35 % [2,19,39]. Digestate high in moisture content has been reported to displace oxygen in soil when used both as fertilizer and as soil amendment [2,19]. The displacement of oxygen in soil due to high moisture content of digestate used as biofertilizer negatively impacts on the receiving soil by creating anaerobic conditions, which force aerobic microorganisms to use nutrients like N, Mn, and S as a source of energy for their life cycles [2,39]. The N, Mn, and S are crucial macronutrients for root growth and cytokine synthesis and high moisture present in digestate will reduce their bioavailability in receiving soil [2]. Since the moisture content of digestate reported in this study were observed to exceed the recommended value of <35 %, moisture reduction applications or techniques either thermal or chemical which can contribute to increasing sanitation while retaining the nutritive value for plants and soil improvement capacity are highly recommended [2]. Moisture contents in this study did not differ significantly (p < 0.05) across pre-treated and untreated digestate samples, but pretreatment was observed to increase moisture content in digestates as compared to untreated digestate. This observation was contradictory to the finding of [2,19,39,44], and [44] who reported a reduction in moisture content in pre-treated digestate as compared to untreated digestate samples as higher temperature increases moisture reduction. The increase in moisture contents with decrease in volatile solid and total solid values in digestate was reported to be optimal characteristics of a stable digestate produced during AD [[45], [46], [47]] and similar trends were observed in this study.

Ammonia-nitrogen, which is an inorganic substance present in mineral form, is not consumed by anaerobes during AD, thus its presence makes digestate suitable to be used as biofertilizer [45,46]. The results for NH_4_^+^-N in this study ranged from 50.09 ± 2.19 to 67.09 ± 2.94 % TKN across analyzed digestate samples and while TKN ranged from 47.32 ± 2.03 g/kg to 57.33 ± 2.36 g/kg (Table 1). The results obtained in this study indicate that the most nitrogen contained in red meat abattoir digestate analyzed in this study is available in mineral form as NH_4_^+^-N as it represent more than 50 % of the total nitrogen. The quantity of nitrogen in digestate suggest that digestate is the nitrogen-based fertilizer in ammonium-N form [19,45]. Studies conducted by Refs. [45,46], have stated that the predominance of nitrogen in mineral form enhance the potential of digestate to be utilized as biofertilizer and while on the other hand reduces its use as soil amendment. Thus, results observed in this study are in support of the findings reported by Refs. [2,19,43,45], and [46], the digestate is a by-product rich in NH_4_^+^-N in mineral form.

The presence of NH_4_^+^-N in high concentration in digestates increases its biofertilizer use in agricultural soil as plants can easily recognize it as nitrogen nutrient source. In digestates with a pH around 7.5 to 8.5, the unionized NH_4_^+^-N form is the most abundant form of nitrogen. This is evident in this study as the pH reported is within the pH range. Pasteurized and sterilized samples in this study were observed to consist of higher concentration of NH_4_^+^-N as compared to untreated sample. This may be attributed to high temperature enhancing the biodegradation of protein contents within the abattoir feedstock by microorganism [45]. The results for NH_4_^+^-N differed significantly (p < 0.05) across pretreatments and untreated digestate. No significant difference (p < 0.05) was observed between sterilized and pasteurized digestate samples for results obtained for NH_4_^+^-N concentrations. The higher ammonium-nitrogen in pre-treated samples as compared to untreated digestate samples can be attributed to the high pH and temperature which has been reported to effectively increase the release of NH_4_^+^-N during AD [2,38,45,46].

EC is a very important parameter, as it is used to determine the applicability of digestate on soil in order to prevent high salinity that will negatively affect soil health and plant growth. The EC values in this study for abattoir digestate ranged from 1759 ± 83.33 μS/cm to 3334 ± 35.33 μS/cm across analyzed samples and the results differed significantly (p < 0.05) amongst treatments (Table 1). The EC results in this study fell within the recommended value of <3000 μS/cm stipulated by Ref. [38], except for PSW (untreated) which was observed to reach as high as 3334 ± 35.33 μS/cm. Previous studies [19,26,[42], [43], [44]] has shown that the use of digestate with EC exceeding a threshold of 3000 μS/cm can cause osmotic stress which results in ultimate lower productivity of crops. Results in this study were observed to be lower than the required threshold thus indicating that adverse osmotic stress in plants may not occur.

The result for COD in this study ranged from 1441.35 ± 178.08 mg/L to 3873.41 ± 399.17 mg/L across abattoir digestate samples and the results differed significantly (p < 0.05) amongst treatments for cattle, sheep, and pig abattoir digestate (Table 1). Results for COD in this study showed a major reduction of COD in the pretreated digestate samples as compared to untreated digestate samples. According to Ref. [14], these high decrease in COD of pre-treated digestate samples as compared to untreated digestate samples may be attributed to organic matter consumption by relevant microflora during its biological activity progression in the digesters.

### 3.2 Results for VFA concentration in red meat abattoir digestate

VFA's such as acetic acid and propionic acid with concertation >300 mg L^−1^ COD have been reported in previous studies to exhibit characteristics opposing phytopathogenic microorganisms [2,19]. They also reported that the mortality of these microorganisms occurs in just seconds because of the modified osmotic gradient of the cell membrane exhibited by presence of VFA's above the mentioned concertation value. The results for TVFA in this study ranged from 2102.27 ± 398.21 to 3230.82 ± 421.89 mg L^−1^ COD (Table 1) and were observed to be below the threshold of about as recommended by Ref. [38]. These can be attributed to the active consumption of VFAs by methanogens during AD.

**Fig. 1:**
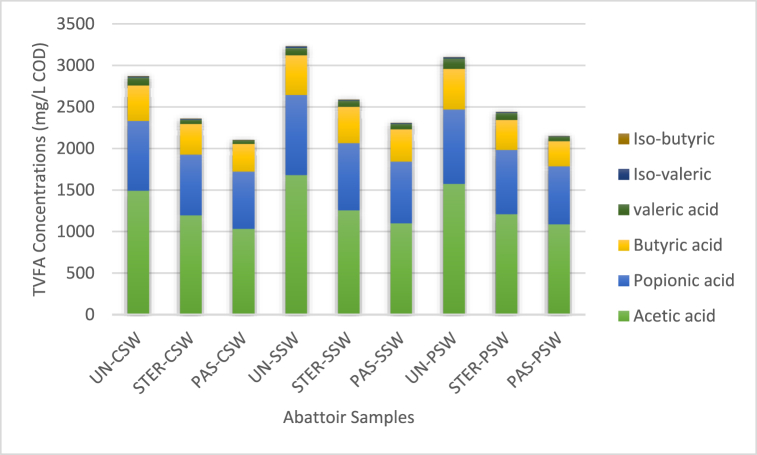
TVFA composition of untreated, sterilized, and pasteurized cattle, sheep, and pig anaerobically digested high throughput red meat abattoir waste analyzed at the end of AD process (day 40). Graph represents means of TVFAs of 20 digested sample per red meat animal type for untreated, sterilized, and pasteurized red meat abattoir waste digestate. (For interpretation of the references to colour in this figure legend, the reader is referred to the Web version of this article.)

In this study, the most prominent VFA were acetic acid and propionic acid followed by butyric acid in all digestate samples with concentrations ranging from 1032 ± 89.33 to 1681.09 ± 109.11 mg L^−1^ COD, 690 ± 56.37 to 964.53 ± 67.05 mg L^−1^ COD, and 300.84 ± 77.28 to 486.13 ± 69.47 mg L^−1^ COD for cattle, sheep and pig abattoir digested samples respectively (Fig. 1). The least VFA detected in all digestate samples were Iso-valeric acid and Iso-butyric acid with concentration ranging from 1.56 ± 0.27 16.50 ± 1.09 mg L^−1^ COD and 0.44 ± 0.06 to 2.77 ± 0.37 mg L^−1^ COD for cattle, sheep and pig abattoir digested samples respectively (Fig. 1). High concentration of acetic acid and propionic acids observed in this study indicates that the presence of this acids in digestate enhances the sanitation of digestate as they have the ability to inactivate indicator pathogens present (*E.coli, Salmonella, Listeria* etc.) in red meat abattoir waste feedstock during AD.

### 3.3 Results of macro- and micronutrients concentrations in red meat abattoir digestate

Digestate has been reported in literature to contain both macro- and micro-nutrients [47]. Macro and micro-nutrients present in substrate are not affected by AD but are conserved and transformed to a more organic form which is readily available to crops. During their study [47] noted that concentration of micronutrients and macronutrients such as N, P and K in substrates being fed to the digester is equivalent to the concentration in the digestate. The results for micro and macro elements composition of red meat abattoir waste digestate are represented in Table 2. When digestate is recycled to land as a biofertilizer, most of these micronutrients are fully utilized, as they are essential for plant and microbial growth. Phosphorus, N, Mg, Ca, S and K are essential elements for plant growth and the determination of their concentration in digestate is of great importance for its sustainability as biofertilizer [23,48]. These essential elements were observed to be the most abundant in all analyzed digestate samples in this study, thus providing further evidence on the potential use of digestate derived from AD of red meat abattoir waste as biofertilizer for agricultural purposes. The N, P, K contribute most to the fertilizing properties of organic soil amendment as these are the primary plant nutrients which are required in great quantities as compared to other secondary plant nutrients [49,50]. This was also observed in this study as P and K ranged from 12.4 to 27.1 g/kg and 27.2–57.7 g/kg respectively except for N as the TKN (>47 g/kg in all analyzed digestate samples) observed in this study was in mineral form as NH_4_^+^-N.

**Table 2:** The micro and macro elements composition of untreated, sterilized, and pasteurized cattle, sheep, and pig anaerobically digested high throughput red meat abattoir waste analyzed at the end of AD process. Results are means ± standard deviation of 20 digested sample per red meat animal type for untreated, sterilized and pasteurized red meat abattoir digestate.

Parameter	Unit	Abattoir Digestate Results (mean ± SD)								
		CSW (untreated)	CSW (sterilized)	CSW (Pasteurized)	SSW (untreated)	SSW (sterilized)	SSW (pasteurized)	PSW (untreated)	PSW (sterilized)	PSW (pasteurized)
Ca	g/Kg	30.9 ± 0.92	27.4 ± 0.73	25.7 ± 0.90	27.31 ± 0.48	19.66 ± 0.34	19.52 ± 0.46	12.3 ± 0.56	10.3 ± 0.94	10.3 ± 0.67
Mg	g/Kg	2.41 ± 0.16	1.95 ± 0.22	1.75 ± 0.09	2.33 ± 0.21	1.67 ± 0.17	1.46 ± 0.12	2.36 ± 0.29	1.88 ± 0.17	1.43 ± 0.14
K	g/Kg	57.7 ± 0.65	49.6 ± 1.09	35.5 ± 0.77	44.15 ± 1.22	37.12 ± 0.98	36.08 ± 0.67	33.4 ± 0.84	27.2 ± 1.03	28.6 ± 0.77
Na	g/Kg	14.8 ± 0.29	13.0 ± 0.38	8.86 ± 0.17	12.31 ± 0.36	10.64 ± 0.33	9.73 ± 0.17	10.71 ± 0.49	9.12 ± 0.21	9.12 ± 0.30
S	mg/Kg	600.37 ± 5.56	412.33 ± 4.96	409.67 ± 3.49	548.91 ± 7.05	430.26 ± 3.05	429.81 ± 2.20	566.08 ± 5.72	359.05 ± 4.53	362.33 ± 3.81
P	g/Kg	27.1 ± 0.38	20.7 ± 0.49	19.2 ± 0.31	23.11 ± 0.63	16.23 ± 0.51	15.46 ± 0.66	15.9 ± 0.59	12.4 ± 0.31	13.5 ± 0.41
Fe	g/Kg	7.67 ± 0.34	5.20 ± 0.24	4.66 ± 0.19	5.93 ± 0.41	4.27 ± 0.36	4.13 ± 0.39	4.71 ± 0.56	3.88 ± 0.39	3.86 ± 0.26
Mn	mg/Kg	128.11 ± 1.82	96.07 ± 1.23	93.41 ± 0.83	139.22 ± 2.53	120.09 ± 1.15	119.32 ± 0.99	149.31 ± 2.01	125.09 ± 1.31	123.14 ± 1.55
B	mg/Kg	35.3 ± 0.78	29.0 ± 0.64	28.6 ± 0.52	26.94 ± 0.86	20.19 ± 0.40	18.71 ± 0.45	15.5 ± 0.69	12.2 ± 0.37	12.8 ± 0.33
Mo	mg/Kg	1.26 ± 0.03	0.47 ± 0.04	0.44 ± 0.06	1.33 ± 0.16	0.53 ± 0.04	0.57 ± 0.05	1.45 ± 0.09	0.67 ± 0.07	0.62 ± 0.04
Al	mg/Kg	232.09 ± 0.35	127.72 ± 0.84	124.86 ± 0.53	267.14 ± 0.67	181.27 ± 0.74	175.33 ± 049	303.05 ± 1.01	213.54 ± 0.87	209.33 ± 0.79
N-org	%TKN	3.23 ± 0.07	2.89 ± 0.05	2.47 ± 0.09	4.21 ± 0.05	3.19 ± 0.03	2.97 ± 0.08	3.92 ± 0.06	3.08 ± 0.07	2.87 ± 0.03
C-org	%	34.1 ± 0.43	33.1 ± 0.21	33.4 ± 0.18	40.69 ± 0.34	37.08 ± 0.39	35.84 ± 0.22	42.7 ± 0.64	36.6 ± 0.51	32.6 ± 0.47
C/N		8.06 ± 0.13	11.45 ± 0.08	13.52 ± 0.12	9.67 ± 0.21	11.62 ± 0.17	12.07 ± 0.31	10.89 ± 0.29	11.79 ± 0.35	11.36 ± 0.58

CSW = cattle slaughter waste; SSW = sheep slaughter waste; PSW = pig slaughter waste.

The regulation of [38] stated the concentrations for micro and macronutrients (Ca, Mg, K, Na, S, P, Fe, Mn, B, Mo, Al) need to be provided for application purposes of digestate in agricultural fields and there are no set standard limits on their abundance in digestate. The digestate's P content stimulates the development of roots, reproduction, the stiffness of the tissues and the quality of plant products. The high amount of P and K and other minerals e.g. Ca, which are readily available to plants, is also attributed to digestate's excellent fertilizing potential. Ca is an essential plant nutrient, which is responsible for cell wall formation and cell membrane. Ca is also responsible for growth promotion of roots and stem, it is responsible for plant rigidity and vigour. The results for Ca were observed to range from 10.3 ± 0.94–30.9 ± 0.92 g/kg (Table 2), thus indicating the benefit digestate will impose on stimulation of root development, reproduction of roots and plant development when valorized as biofertilizer.

The results for Na, Mg, and Fe ranged from 8.86 to 14.8 g/kg, 1.46–2.41g/Kg, and 3.88–7.67 g/kg respectively across studied samples (Table 2). S, Mn, B, Mo, and Al were less abundant in all abattoir waste digestate samples tested in this study and the results differed significantly (p < 0.05). Their concentration ranged from 259.05 to 600.37 mg/kg, 93–149 mg/kg, 12.2–35.3 mg/kg, 0.44–1.45 mg/kg and 175.33–303.05 mg/kg respectively across studied abattoir digestate samples. The presence of Fe, Mn, B, Mo and Al in digestate favor the digestate as a biofertilizer as these nutrients have been reported to be responsible for enzyme activation, promote microbial activity in soil and chlorophyll formation in plants [31,45]. The Fe, Mn, B, Mo and Al were detected in all analyzed digestate in this study thus further indicating that digestate from AD red meat abattoir may also contribute to promoting enzyme activation, enhance microbial activity in receiving soil and promote chlorophyll formation when valorized as biofertilizer.

Carbon and nitrogen concentrations are vital in digestate as they influence its agronomic use. Being the crucial constituent of amino acids, nitrogen is essential for the plant's growth and is also a crucial nutrient for photosynthesis [45,51]. Nitrogen and carbon contents in abattoir waste digestate in this study ranged from 3.92 to 5.19 % and 33.1–47.08 % respectively and the results differed significantly (p < 0.05) between treated abattoir digestate samples. According to this, abattoir digestate is nutrient-rich and may improve soil microbial and nutrient status when utilized as biofertilizer, especially in nutrient-depleted soils [45].

### 3.4 Results for E. coli, Listeria and Salmonella concentration for abattoir digestate

Results for the studied microbial concentrations (*E. coli, Listeria and Salmonella),* are presented in Table 3 and [38] was used as a benchmark for quality control for digestate intended to be valorized a biofertilizer. Microbial toxins emanating from inadequately treated digestates may pollute agricultural lands when utilized as biofertilizer and/or soil conditioner [39,52,53].

**Table 3:** Microbial composition of untreated, sterilized, and pasteurized cattle, sheep, and pig anaerobically digested high throughput red meat abattoir waste analyzed at the end of AD process. Results are means ± standard deviation of 20 digested sample per red meat animal type for untreated sterilized and pasteurized red meat digestate and results were benchmarked with PAS110 for quality control of digestate.

Parameter	Unit	Abattoir Digestate Results (mean ± SD)									PAS 110
		CSW-D(untreated)	CSW-D (sterilized)	CSW-D(Pasteurized)	SSW-D (untreated)	SSW-D (sterilized)	SSW-D (pasteurized)	PSW-D (untreated)	PSW-D(sterilized)	PSW-D (pasteurized)	Upper limits
*E.coli*	cfu/mL	1023 ± 35	873 ± 22	715 ± 31	997 ± 45	800 ± 19	694 ± 23	1068 ± 51	933 ± 37	733 ± 44	<1000 cfu/mL of fresh sample
*Salmonella*	25g of fresh sample	ND	ND	ND	ND	ND	ND	ND	ND	ND	Absent in 25g of fresh sample
*Listeria*	25g of fresh sample	ND	ND	ND	ND	ND	ND	ND	ND	ND	Absent in 25g of fresh sample

ND = not detected; CSW-D = cattle slaughter waste digestate; SSW-D = sheep slaughter waste digestate; PSW-D = pig slaughter waste digestate; SD = standard deviation.

Results for *E. coli* in this study ranged from 694 ± 23 cfu/mL to 1068 ± 51 cfu/mL and the results differed significantly (p < 0.05) amongst treatments in cattle, sheep and pig abattoir digestate. The results observed for *E. coli* detection in this study fell within the [38] limit except for results obtained for untreated digestate for cattle and pig digestate where values of about 1023 ± 35 cfu/mL and 1068 ± 51 cfu/mL were observed respectively (Table 3). This may be attributed to the high temperature treatment that was used to pre-treat the red meat abattoir waste feedstock. *Salmonella* sp. and *Listeria* sp. were not detected in all abattoir digestates in this study and the results fell within the recommended by Ref. [38] standard limit. According to a study by Ref. [9], 90 % of pathogenic bacteria reduction is archived when AD is conducted at HRT >20 days and mesophilic temperature of 37 °C. Similar trends observed by Ref. [9] were also observed in this study whereby HRT was 40 days and AD process kept at 37 °C. Ammonium-nitrogen has been reported to be phytotoxic to pathogens during AD which can also be attributed to the non-detection of Salmonella and listeria. Indicator pathogens such as *E. coli*, *Salmonella*, and *Listeria* are gram negative species which are sensitive to thermophilic temperature, high pH > 6.5, VFA >350 mg/L, and NH_4_^+^-N which causes complete inactivation of these pathogens. This was also observed in this study as pretreatment of red meat abattoir feedstock prior to AD was conducted using pasteurization at 70 °C for 1 h and sterilization at 133 °C at 3 bars for 15 min, while pH and VFA concentrations were >6 and > 350 mg/L resulted in non-detection of pathogenic microorganisms. Acetic acid and propionic acid with concertation >300 mg/L COD were observed by Refs. [2,19] to exhibit phytopathogenic microorganisms and similar observations were noted where the concentration of both acetic acid and propionic acid was >600 mg/L COD in all tested digestate samples. These findings of inactivation of pathogenic microbial in this study indicate that AD treatment of red meat abattoir waste produced a sanitized product that will not affect the environment with microbial contamination when valorized as biofertilizer.

### 3.5 Results for heavy metal concentration analyzed in red meat abattoir digestate

The results for heavy metals concentrations are presented in Table 4, and [38] was used as benchmark reference in this study. The concentration of toxic metals in the digestate does not only pose a threat to the receiving soils and its harmful effects are not only limited to soil and plant microbes such as nitrogen-fixing bacteria, but can also impact humans and animals indirectly through food ingestion grown by using digestate as biofertilizer. Since As metals such as Ni, Zn, and Cu are mostly present in animal feeds for stimulate growth and as antimicrobials, their presence in digestate cannot be excluded. Ni, Zn, and Cu are also trace elements that are essential for the biological processes in plants.

**Table 4:** Heavy metal concentration composition of untreated, sterilized, and pasteurized cattle, sheep, and pig anaerobically digested high throughput red meat abattoir waste. Results presents mean ± standard deviation of 20 digested sample per red meat animal type for untreated, sterilized, and pasteurized red meat digestate and results were benchmarked with PAS110 for quality control of digestate.

Parameter	Unit	Results (mean ± SD)									PAS 110
		CSW (untreated)	CSW (sterilized)	CSW (Pasteurized)	SSW (untreated)	SSW (sterilized)	SSW (pasteurized)	PSW (untreated)	PSW (sterilized)	PSW (pasteurized)	Upper limits
Zn	mg/Kg	273 ± 7.21	196 ± 4.36	162.26 ± 5.51	269.16 ± 4.58	127.88 ± 6.51	120.49 ± 2.52	188.19 ± 3.57	80.35 ± 4.17	98.08 ± 3.66	400 mg/kg
Cu	Mg/Kg	48.5 ± 1.90	36.72 ± 1.09	35.8 ± 0.99	56.73 ± 2.04	43.57 ± 1.33	41.88 ± 0.87	65.0 ± 1.05	53.61 ± 1.33	55.53 ± 1.01	200 mg/Kg
Cd	mg/Kg	0.89 ± 0.04	0.61 ± 0.06	0.57 ± 0.03	1.01 ± 0.05	0.77 ± 0.08	0.71 ± 0.04	0.83 ± 0.06	0.66 ± 0.05	0.62 ± 0.03	1.5 mg/Kg
Cr	mg/Kg	43.59 ± 2.69	29.33 ± 1.66	19.05 ± 1.14	38.79 ± 1.87	23.55 ± 0.99	20.19 ± 1.01	36.91 ± 1.53	24.62 ± 1.17	21.95 ± 1.09	100 mg/Kg
Pb	mg/Kg	10.23 ± 0.71	4.63 ± 0.45	3.97 ± 0.31	11.09 ± 0.65	5.23 ± 0.58	4.75 ± 0.27	9.56 ± 0.77	4.42 ± 0.58	4.12 ± 0.39	200 mg/Kg
Ni	mg/Kg	3.08 ± 0.04	1.39 ± 0.07	1.30 ± 0.04	3.22 ± 0.07	1.62 ± 0.04	1.59 ± 0.03	4.23 ± 0.06	2.26 ± 0.05	2.16 ± 0.07	50 mg/kg
Hg	mg/Kg	0.08 ± 0.01	0.00 ± 0.00	0.00 ± 0.00	0.07 ± 0.00	0 ± 0.00	0 ± 0.00	0.09 ± 0.01	0.00 ± 0.00	0 ± 0.00	1 mg/Kg
As	mg/Kg	4.93 ± 0.30	2.47 ± 0.19	1.99 ± 0.34	5.87 ± 0.41	5.23 ± 0.22	4.65 ± 0.38	4.93 ± 0.45	2.74 ± 0.33	2.09 ± 0.29	40 mg/Kg

ND = not detected; CSW = cattle slaughter waste; SSW= Sheep waste; PSW = pig slaughter waste; SD = standard deviation.

Since anaerobic digestion cannot eliminate heavy metals completely, their presence may pose a threat and possible environmental contamination. To gain confidence in utilizing digestate as biofertilizer [38], has set limits of heavy metals in digestate that will be valorized in agricultural lands and as soil conditioners. To ensure that the buildup of heavy metals in agricultural soils is below any levels that could adversely impact plant development and crop yield, as well as animals and humans who eat them, these regulatory requirements were developed to safeguard the environment, animals and humans [39,53].

The concentration of Zn, Cu, Cd and Cr ranged from 80.35 ± 4.17 to 273 ± 7.21 mg/kg, 35.8 ± 0.99 to 65.0 ± 1.05 mg/kg, 0.57 ± 0.03 to 1.01 ± 0.05 mg/kg, 19.05 ± 1.14 to 43.59 ± 2.69 mg/kg across studied abattoir digestate samples (Table 4). The concentration of Pb, Ni, Hg and As ranged from 3.97 ± 0.31 to 11.09 ± 0.65 mg/L, 1.30 ± 0.04 to 4.23 ± 0.06 mg/kg, 0.00 ± 0.00 to 0.09 ± 0.01 mg/kg and1.99 ± 0.34 to 5.87 ± 0.41 across studied abattoir digestate samples (Table 4). The results obtained in this study for the heavy metals concentrations in abattoir digestate were within the limits as recommended by Ref. [38] as safe levels for heavy metals within a biofertilizer, and the concentrations did not differ significantly (p < 0.05) for sterilized and pasteurized pretreated abattoir digestate samples. The most predominant heavy metal present in all tested digestate in this study was Zn, even though it was below the detection limit of about <400 mg/kg [38] in all samples. This can be attributed to its high content in animal feed [39,52]. Mercury was the only heavy metal that not detected in all pasteurized and sterilized abattoir waste digestate samples in this study. This may be attributed to the volatilization of this element during pretreatment of red meat abattoir waste [19,39,52]. The concentrations observed in this study across all studied abattoir digestate are favorable for digestate use as biofertilizer as Hg has been reported to be harmful at low concentration (4 mg in soil and 2 mg in plants) due to its bioaccumulation and high toxicity in soil and plants. The presence of Hg in digestate in concentration higher that 1 mg have been reported to inactivate Sulphur thus causing inhibition enzyme activities vital for soil health and plant growth. Hg has been reported to cause severe neurological disorders in humans and animals even if ingested in low concentrations. The presence of Hg heavy metals in digestate renders it unfavorable to be utilized as biofertilizer as can affect the health of animals and humans as a result of consuming the plants grown using it as fertilizer. In this study high temperature due to pre-treatments was observed to volatize Hg and other tested heavy metals thus increasing the sanitary quality of digestate thus giving more confidence in its use as biofertilizer on agricultural lands. Therefore, it may be concluded that the use of these red meat abattoir digestates in agriculture is not expected to cause metal related adverse effects on the environment.

## 4 Conclusion

The physicochemical characteristics of abattoir waste derived digestate and its potential use as biofertilizer revealed that abattoir digestate has a potential to be utilized as biofertilizer. Pasteurized pre-treated abattoir digestate was shown to be more stable as compared to sterilized pre-treated and untreated abattoir digestate samples. Pasteurized CSW digestate has been observed to be more stable as compared to other digestate samples in this study. Moisture values reported in this study for all abattoir digestates exceeded the PAS 110 which recommended value < 35 % in digestates that will be used as biofertilizer, thus post treatment of digestate either by thermal or chemical treatment is recommended to reduce moisture in digestate. This will also in turn contributes to enhancing digestate sanitation without impacting on its soil improvement capacities. Because of the high nutritional content (N, P, and K) that is readily bioavailable to plants and the removal/reduction of toxins like pathogens and heavy metals, the AD of abattoir waste enabled generating a final product (digestate) with very good fertilizing qualities. The results obtained in this study for abattoir digestate met the PAS 110 quality standards and indicates that it has a great potential to be utilized as a biofertilizer. The pasteurized CSW was observed to be the most stable as it yielded better results (e.g. lower heavy metal and microbial concentrations) as compared to other red meat abattoir digestates analyzed. In light of this, the digestate seems to be a very good competitor to replace the application of inorganic fertilizers. The findings of this study are key to the contribution towards abattoir waste valorization and development of guidelines by policy makers for the application of digestate derived from abattoir slaughter waste in South African agricultural land. It should be noted that comparison of data in this study with available literature data to check similarities were limited due to the limited studies available on AD of red meat abattoir slaughter solid was worldwide. It is therefore recommended that more research on utilizing red meat abattoir slaughter solid waste as feed stock for AD and characterizing the resultant digestate should be encouraged to broaden the literature and fill the knowledge gap in both developing and developed countries.

## Funding statement

The study was funded by 10.13039/100004420United Nations Industrial Development Organization (UNIDO) and the Agricultural Research Council-Animal Production.

## Data availability statement

Data included in article/supp. material/referenced in article.

## Additional information

No additional information is available for this paper.

## Declaration of competing interest

The authors declare the following financial interests/personal relationships which may be considered as potential competing interests:

Dr. Mary-Jane Thaela-Chimuka reports financial support, equipment, supplies, and travel were provided by 10.13039/501100001321National Research Foundation (NRF). Dr. Mary-Jane Thaela-Chimuka reports financial support, equipment, supplies, and travel were provided by United Nations Industrial Development Organization (UNIDO).
